# Electrochemical Behavior and Determination of Chlorogenic Acid Based on Multi-Walled Carbon Nanotubes Modified Screen-Printed Electrode

**DOI:** 10.3390/s16111797

**Published:** 2016-10-27

**Authors:** Xiaoyan Ma, Hongqiao Yang, Huabin Xiong, Xiaofen Li, Jinting Gao, Yuntao Gao

**Affiliations:** 1 The Engineering Laboratory of Polylactic Acid-Based Functional Materials of Yunnan, School of Chemistry and Environment, Yunnan Minzu University, Kunming 650500, China; 18788481659@163.com (X.M.); yanghqiao2010227@163.com (H.Y.); xionghuabin@ynni.edu.cn (H.X.); wjplxf24@163.com (X.L.); 15198774152@163.com (J.G.); 2 Key Laboratory of Chemistry in Ethnic Medicinal Resources, State Ethnic Affairs Commission & Ministry of Education, Yunnan Minzu University, Kunming 650500, China

**Keywords:** chlorogenic acid, screen-printed electrode, multi-walled carbon nanotubes

## Abstract

In this paper, the multi-walled carbon nanotubes modified screen-printed electrode (MWCNTs/SPE) was prepared and the MWCNTs/SPE was employed for the electrochemical determination of the antioxidant substance chlorogenic acids (CGAs). A pair of well-defined redox peaks of CGA was observed at the MWCNTs/SPE in 0.10 mol/L acetic acid-sodium acetate buffer (pH 6.2) and the electrode process was adsorption-controlled. Cyclic voltammetry (CV) and differential pulse voltammetry (DPV) methods for the determination of CGA were proposed based on the MWCNTs/SPE. Under the optimal conditions, the proposed method exhibited linear ranges from 0.17 to 15.8 µg/mL, and the linear regression equation was Ipa (µA) = 4.1993 C (×10^−5^ mol/L) + 1.1039 (r = 0.9976) and the detection limit for CGA could reach 0.12 µg/mL. The recovery of matrine was 94.74%–106.65% (RSD = 2.92%) in coffee beans. The proposed method is quick, sensitive, reliable, and can be used for the determination of CGA.

## 1. Introduction

**Figure 1:**
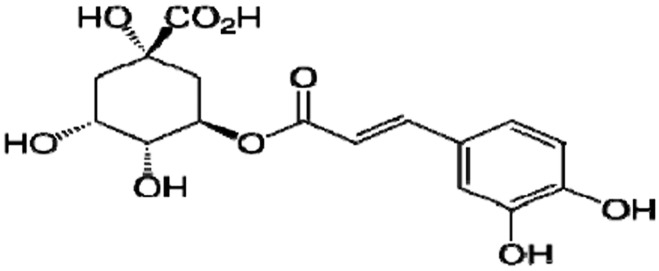
The structure of chlorogenic acid (CGA).

Chlorogenic acids (CGAs) (Figure 1) are a group of polyphenolic compounds common in different plant materials including many common foods and beverages [1], but especially in coffee, which has one of the highest concentrations of CGA of all plant constituents [2]. Many reports have indicated that a diet rich in CGA compounds plays a significant role in preventing many negative effects of aging, as well various diseases associated with oxidative stress such as cancer, cardiovascular, aging and neurodegenerative disease [3,4].

Several methods have been developed for the determination of chlorogenic acid and its derivatives in coffee beans and other plants. The most widely used methods are HPLC [5,6,7], capillary electrophoretic [8,9], and Miceller electrokinetic chromatography [10]. Although these developed methods have been efficient for the quantification of CGA and its derivatives, they have been criticized as being tedious and time consuming, and most of the instruments necessary for these methods are very expensive. In addition, UV-Vis spectrophotometer method is simple, fast and inexpensive for the determination of CGA in coffee beans; however, a direct spectral determination in coffee beans is relatively difficult, because of the spectral overlap with caffeine.

In recent years, electrochemical methods have been widely investigated in the determination of phenolic compounds due to their simplicity, low cost, high sensitivity and rapid response [11,12]. Furthermore, caffeic acid as another important component of coffee can be broadly studied by cyclic voltammetry methods [13,14]. Nevertheless, to the best of our knowledge, the electrochemical determination of CGA has barely been reported.

Screen-printed electrodes (SPEs) are especially recommended in the large-scale production of electrodes with easy-use and portability properties, which have been studied by Hart, Banks and Wang [15,16,17,18]. Also, these miniaturized screen-printed electrodes are suitable for working with sample microvolumes, and are disposable [19,20]. Screen-printed electrodes modified with multi-walled carbon nanotubes (MWCNTs/SPE) improve electron transfer properties, resulting in high sensitivity and low detection limits, decreased overpotentials, ease of mass production, and practicality [21]. Furthermore, they are described as useful electroanalytical tools for the development of analytical applications [22,23,24,25].

In this paper, we applied a simple and fast way to detect CGA with a highly sensitive voltammetric analysis method by using a modified screen-printed electrode with multi-walled carbon nanotubes. Here, the multi-walled carbon nanotubes material with a modified screen-printed electrode was prepared. The electrochemical behavior of CGA at MWCNTs/SPE was investigated, and a sensitive electrochemical analysis method of differential pulse voltammetry (DPV) was developed for the determination of CGA. Furthermore, the proposed method can be used in the quantitative determination of CGA in coffee beans.

## 2. Materials and Methods 

### 2.1. Instruments, Materials and Reagents

All electrochemical experiments were conducted with a ZAHNER Zennium IM6 Electrochemical Workstation (ZAHNER-elektrik GmbH and Co. KG, Kronach, Germany) with an integrated screen-printed three electrode device: a carbon working electrode, a carbon counter electrode, and an Ag/AgCl reference electrode. Scanning electron microscope (SEM, JSM-6360LV, JEOL, Co., Ltd., Tokyo, Japan).

The carboxyl functionalized multi-walled carbon nanotubes (MWCNTs, purity > 95%, with a diameter of 10 nanometers, length of 5 nm) were purchased from Chengdu Institute of Organic Chemistry, Chinese Academy of Sciences; The screen-printed electrodes(work area of 3.1 square millimeter) were purchased from Methrom, Co., Ltd., Beijing, China; Coffee beans were sourced from Puèr University, Puèr, China; The specific concentration of chlorogenic acid (purity > 98%) was purchased from Sigma, St. Louis, MO, USA, and saved at 4 °C. 0.10 mol/L sodium hydrogen phosphate-potassium dihydrogen phosphate buffer, 0.10 mol/L phosphate buffer solution (PBS), 0.10 mol/L citric acid buffer, 0.10 mol/L acetic acid-sodium acetate buffer, 0.10 mol/L sodium hydroxide solution, and 0.50 mmol/L potassium ferricyanide -potassium ferrocyanide solution (K_3_Fe(CN)_6_-K_4_Fe(CN)_6_).

Other reagents used were of analytical-reagent grade. Twice-distilled water was used throughout all experiments.

### 2.2. Experimental Methods

#### 2.2.1. Purification and Functionalization of the Multi-Walled Carbon Nanotubes

**Figure 2:**
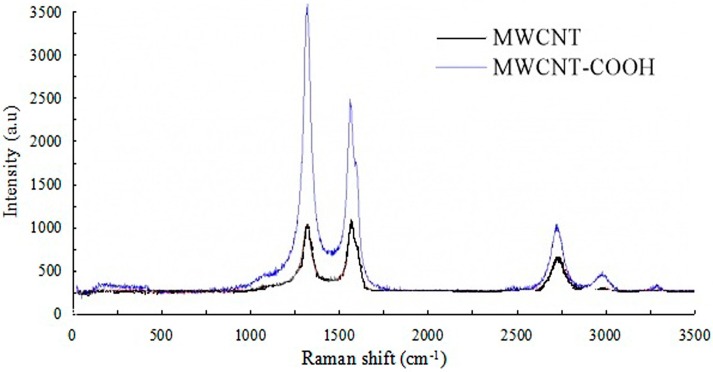
Raman spectra of multi-walled carbon nanotube (MWCNT) and MWCNT-COOH.

In a 100 mL, three-necked, round-bottomed flask, the multi-walled carbon nanotubes of 500 mg and 50 mL concentrated nitric acid were firstly added and mixed homogenously. Next, the mixture was constant-temperature reflowed for 12 h at 140 °C in an oil bath. Then, it was separated in the centrifugal separator. Finally, the multi-walled carbon nanotubes, after centrifugal separation, were washed with distilled water and then dried in a vacuum oven [26]. Thus, the carboxylated multi-walled carbon nanotubes (MWCNT-COOH) were obtained. And the Raman spectra of MWCNT and MWCNT-COOH were shown in Figure 2. Both of the spectra have the same pattern. Moreover, both Raman spectroscopy analyses showed a strong band at 1580 cm^−1^ (G lines) which is the Raman-allowed phonon high-frequency E_2_*_g_* first-order mode, and a disordered-induced peak at 1358 cm^−1^ (D lines), which may originate from defects in the curved graphene sheets, tube ends, as well as the turbostratic structure of graphene in the materials [27,28]. Comparing the ratio of I_G_/I_D_ of the two samples, which are 0.95 for MWCNT, 0.71 for MWCNT-COOH, respectively, we were able to determine that the degree of disorder is reduced after carboxylation. Thus the carboxylation of MWCNTs might improve the electrochemical properties.

#### 2.2.2. Preparation and Characteration of the Multi-Walled Carbon Nanotubes Modified Screen-Printed Electrode

**Figure 3:**
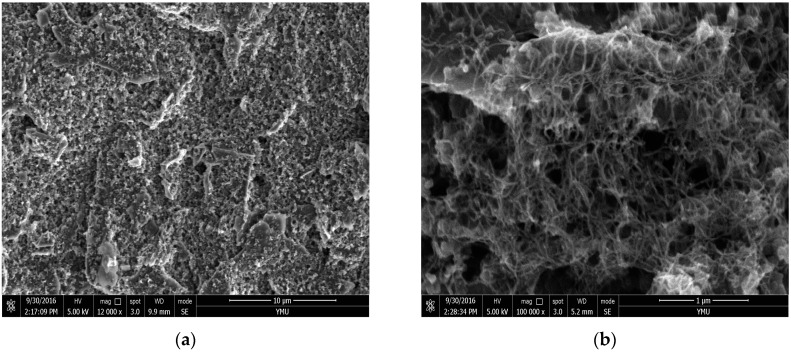
The SEM comparasion of bare screen-printed electrode (SPE) (**a**) and the multi-walled carbon nanotubes modified screen-printed electrode (**b**).

Before modifying the working electrode at the integrated SPEs, the SPEs were first washed with distilled water and dried by N_2_ stream. Then the SPEs was pre-anodized in a 0.1 M (pH = 7.4) PBS containing 0.1 M KCl by applying an anodic potential of +1.9 V (vs. Ag/AgCl) for 120 s. The MWCNTs/SPEs were prepared by coating 5 µL 0.3 mg/mL of the MWCNTs homogeneous suspension onto the SPEs and then dried at room temperature overnight. All modified electrodes were cleaned by cyclic voltammetric technique between –0.5 and +0.5 V at a scan rate of 50 mV/s in PBS (pH 7.4) until a stable cyclic voltammetric response was obtained, and then rinsed with water and dried under a nitrogen stream [29]. The SEM comparison of bare SPE and the multi-walled carbon nanotubes modified screen-printed electrode was shown in Figure 3. The results showed that the surface of the MWCNTs/SPEs was a kind of reticulate cubic structure. It is obvious that the MWCNTs (with little amorphous carbon impurities) were distributed uniformly on the surface of SPE. The spaghetti-like MWCNTs formed a porous structure. The entangled cross-linked fibrils offered high accessible surface area. 

#### 2.2.3. Electrochemical Analysis 

Before using the MWCNTs/SPEs, they were activated in 0.10 mol/L sodium hydroxide solution between the potential range of −0.5 V and 1.0 V at a scan rate of 10 mV/s, then the MWCNTs/SPEs activated were characterized in 0.50 mmol/L potassium ferricyanide-potassium ferrocyanide solution (K_3_Fe(CN)_6_-K_4_Fe(CN)_6_) at a scan rate of 0.15 V/s.

Cyclic voltammetry (CV) and differential pulse voltammetry (DPV) were performed in the three-electrode cell in 0.10 mol/L acetic acid-sodium acetate buffer solution (pH = 6.2) between the potential range of −0.5 V and +0.5 V at a scan rate of 0.15 V/s. The DPV conditions were: pulse width of 50 ms, pulse amplitude of 180 mV and pulse interval of 50 ms.

## 3. Results and Discussion

### 3.1. Cyclic Voltammetry and Differential Pulse Voltammetry Behaviors of CGA at MWCNTs/SPE

**Figure 4:**
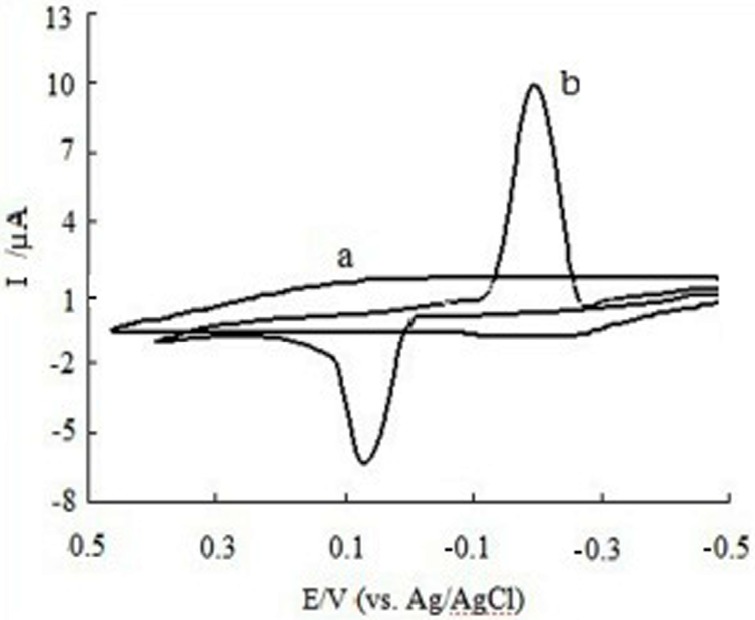
Cyclic voltammetry curves of CGA (**a**) at bare SPE and (**b**) at MWCNTs/SPE.

Figure 4 displays the CV curves of CGA in the 0.10 mol/L acetic acid-sodium acetate buffer solution (pH 6.2) at different electrodes included bare SPE (Figure 4a) and MWCNTs/SPE (Figure 4b). The scan rate is 0.15 V/s with the potential range from −0.5 V to 0.5 V. The result shows that there is no electrochemical response; however, an obvious pair of redox peaks were obtained at MWCNTs/SPE. The oxidation peak potential (E_pa_) and reduction peak potential (E_pc_) of CGA were 0.08 V and −0.19 V (vs. Ag/AgCl), respectively, as ΔE = E_pa_ − E_pc_ = 0.27 V (vs. Ag/AgCl). The ratio of the oxidation peak current and reduction peak (I_pa_:I_pc_) was 0.42, implying that the electrode process of CGA at MWCNTs/SPE is quasi-reversible.

### 3.2. Influence of Supporting Electrolyte and pH

**Figure 5:**
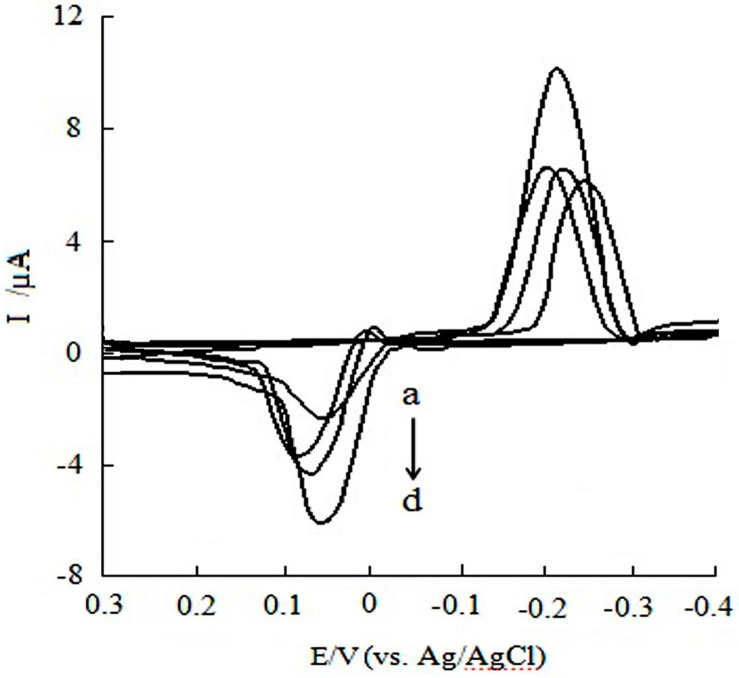
Influence of different supporting electrolytes on peak current: (**a**) 0.10 mol/L potassium hydrogen phosphate-potassium dihydrogen phosphate buffer; (**b**) 0.10 mol/L phosphate buffer solutions; (**c**) 0.10 mol/L citric acid buffer; (**d**) 0.10 mol/L acetic acid-sodium acetate buffer.

Several supporting electrolytes such as 0.10 mol/L potassium hydrogen phosphate-potassium dihydrogen phosphate buffer (Figure 5a), 0.10 mol/L phosphate buffer solutions (PBS, 7% Na_2_HPO_4_ + 1% KH_2_PO_4_ + 90% NaCl + 2% KCl) (Figure 5b), 0.10 mol/L citric acid buffer (Figure 5c), and 0.10 mol/L acetic acid-sodium acetate buffer (Figure 5d) were tested at MWCNTs/SPE. A pair of CV redox peaks are observed in the four supporting electrolytes. A better-defined CV response with higher redox peak of CGA than in the other cases was obtained in 0.10 mol/L acetic acid-sodium acetate buffer.

**Figure 6:**
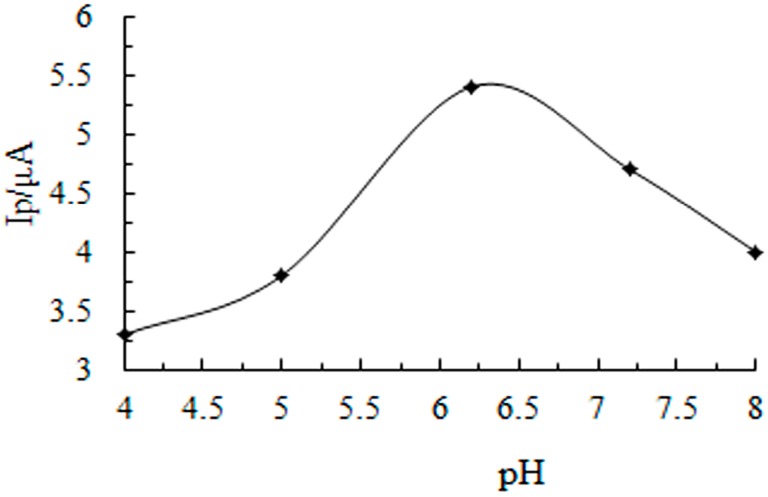
Influence of buffer solution pH to peak current.

The influence of pH was investigated in 0.10 mol/L acetic acid-sodium acetate buffer, as shown in Figure 6, the oxidation peak current of CGA increases with the increasing of pH from 4.0 to 6.2, and then reaches its maximum at pH 6.2, while the oxidation peak current decreases as pH increases above 6.2. Therefore, 0.10 mol/L acetic acid-sodium acetate buffer (pH 6.2) was chosen as the optimal supporting electrolyte for subsequent experiments.

### 3.3. Influence of the Scan Rate

**Figure 7:**
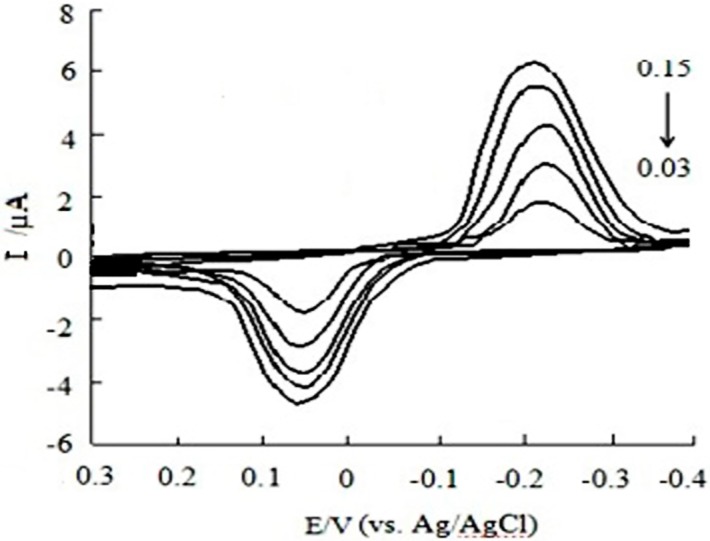
Cyclic voltammetry curves of CGA at different scan rates.

**Figure 8:**
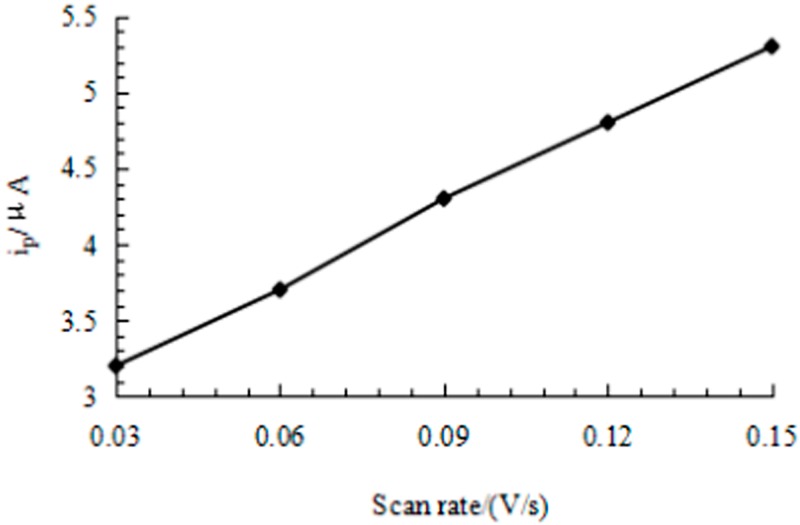
The peak current of CGA at different scan rate.

Figure 7 and Figure 8 shows the effect of scan rate on the CV response of CGA at MWCNTs/SPE in the 0.10 mol/L acetic acid-sodium acetate buffer solution (pH 6.2). It is found that both the oxidation peak current (I_pa_) and reduction peak current (I_pc_) are linear to the scan rate (υ) in the range of 0.03 to 0.15 V/s, the linear regression equations of I_pa_ and I_pc_ are I_pa_ (µA) = 15.887 *ν* + 2.8809 (r = 0.9969) and I_pc_ (µA) = 16.798 *ν* + 4.1028 (r = 0.9955), respectively. This indicates that the I_p_ is proportional to the v but not v^1/2^. Therefore, the electrochemical process of CGA at MWCNTs/SPE is adsorption-controlled. The maximum peak signal-to-noise ratio for CGA was achieved at the scan rate of 0.15 V/s. The scan rate of 0.15 V/s was therefore selected for this work.

The relationship between the peak current (I) and electron transfer number (n) comply with Equation (1) in the electrode reaction according to the Laviron theory [30].

(1) $$I=\frac{n^{2}F^{2}A\Gamma_{T}v}{4\mathrm{RT}}=\frac{\mathrm{nFQv}}{4\mathrm{RT}}$$

While, F (96485 C·mol^−1^), A (cm^2^), Γ_T_ (mol·cm^−2^) and v (mV/s) in the formula are the Faraday constant, the electrode surface area, the adsorption quantity and the scan rate, respectively. Q = nFAΓ_T_, Q is the peak area of a single process of cyclic voltammetry (with quantity of electricity). The oxidation peak electron transfer number（n） was calculated to be 2.01 (v = 0.15 V/s) in this electrode reaction, the oxidation peak potential (E_pa_) and reduction peak potential (E_pc_) of CGA were 0.08 V and −0.19 V (vs. Ag/AgCl), respectively. ΔE = E_pa_ − E_pc_ = 0.27 V (vs. Ag/AgCl), the ratio of the oxidation peak current and reduction peak (I_pa_/I_pc_) was 0.42, implying that the electrode process of CGA at MWCNTs/SPE is quasi-reversible.

### 3.4. The Linear Range and Detection Limit

**Figure 9:**
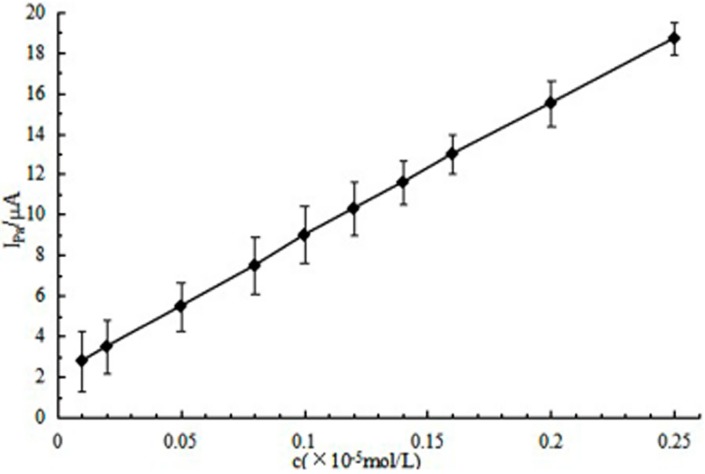
The peak current of CGA at different concentration.

A well-defined oxidation peak DPV responses with a high peak current of CGA was observed at MWCNTs/SPE. MWCNTs/SPE was the working electrode. The different concentrations of CGA were added into acetic acid-sodium acetate buffer solution (pH 6.2), then the differential pulse voltammetry analysis was used in a pulse width of 50 ms, a pulse amplitude of 180 mV, and pulse interval of 50 ms in the potential range of −0.5–0.5 V. We found that the oxidation peak current value was linearly related to the concentration of CGA in the range of 0.17 to 15.8 μg/mL and the detection limit was 0.12 μg/mL (shown in Figure 9). The regression equation was: I_pa_ (µA) = 4.1993 c (10^−5^ mol/L) + 1.1039 (r = 0.9976). 

The detection limit and linear range of the proposed method have been compared with that of the other previously reported methods for the determination of CGA shown in Table 1. It is evident that the proposed electrochemical method shows high sensitivity with the lower detection limit, indicating that MWCNTs/SPE can be used as a sensor for the sensitive electrochemical detection of CGA in many samples [31,32,33,34]. 

### 3.5. Determination of CGA in Green Coffee Bean

The coffee beans (From Puèr University, Puèr, China) were ground to powder with a mortar, and the ground coffee sample was defatted with hexane (1:6; w/v) for 8 h in a Soxhlet extraction system. Then, CGA was extracted from the defatted coffee powder with water using the microwave-assisted extraction (MAE) lab station (Shanghai new apparatus of Microwave Chemical Technology Co., LTD, Shanghai, China) for 5 min under the conditions of 800 w, 50 °C and liquid-solid ratio of 5:1 [35]. According to the proposed method, the MWCNTs/SPE was applied to the determination of CGA in coffee beans, and the result is shown in Table 1. The standard deviation (RSD %) was found to be 1.33%–4.77% and the recovery was 94.74%–106.65%. The result of CGA determination was in good agreement with that specified by HPLC (shown in Table 2.).

## 4. Conclusions

The multi-walled carbon nanotubes material modified screen-printed electrode (MWCNTs/SPE) was prepared and it was applied to the electrochemical behavior research and determination of CGA. The differential pulse voltammetry (DPV) method for the determination of CGA was proposed based on the MWCNTs/SPE. This secured several advantages, as the method is quick, sensitive and reliable. This proposed method can be used for the determination of CGA in the coffee beans. The advantages of this proposed method are: high sensitivity, simplicity of preparation at short time and good reproducibility.

## Figures and Tables

**Table 1:** Comparison of our research with other methods for CGA detection.

Measurement Methods	Linear Range (μg/mL)	Detection Limit (μg/mL)	References
UV-Vis spectroscopy	10.7−39.0	16	[29]
Capillary electrophoresis with chemiluminescence	1.10−110	0.5	[30]
HPLC	0.8−20.0	0.32	[31]
Square-wave voltammetry	1.77−17.7	0.27	[32]
Differential pulse voltammetry (DPV) using a MWCNTs/SPE	0.17−15.8	0.12	this method

**Table 2:** Measurement results of CGA in coffee beans (n = 5).

CGA Sample	By This Method		Added (mg/g)	Found (mg/g)	Recovery (%)	By HPLC * (mg/g)
	(mg/g)	RSD (%)				
1	13.23	4.77	10.00	24.11	106.65	14.71
2	21.12	3.14	10.00	30.01	94.74	19.78
3	25.21	2.47	10.00	36.03	103.25	23.92
4	18.74	1.33	10.00	28.92	100.96	19.03

* The tested conditions of HPLC: The temperature of 25 °C, the flow rate of 1.0 mL/min, the mobile phase was a mixture of acetonitrile (solvent A) and water–glacial acetic acid (99:1, v/v, pH 2.8) (solvent B).
